# Perceptions and Experiences of Frontline Urban Indian Organization Healthcare Workers With Infection Prevention and Control During the COVID-19 Pandemic

**DOI:** 10.3389/fsoc.2021.611961

**Published:** 2021-04-30

**Authors:** Noah Collins, Jolie Crowder, Jamie Ishcomer-Aazami, Dionne Apedjihoun

**Affiliations:** ^1^National Council of Urban Indian Health, Technical Assistance and Research Center, Washington, DC, United States; ^2^International Association for Indigenous Aging, Silver Spring, MD, United States

**Keywords:** public health, mental health, epidemic, COVID-19, healthcare worker, urban Indian health organization, American Indian and Alaska Native, minority health

## Abstract

Coronavirus disease 2019 (COVID-19) has created significant challenges for outpatient healthcare providers and patients across the United States (U.S.). Forty-one Urban Indian Organizations (UIOs), who provide a wide spectrum of health services for American Indian and Alaska Native (AI/AN) populations and other underinsured and uninsured populations in urban areas across the country, are no exception. The National Council of Urban Indian Health (NCUIH), in collaboration with the U.S. Centers for Disease Control and Prevention (CDC), set out to understand the needs, challenges, and opportunities for improvement in infection prevention and control (IPC) training and systems from the perspective of UIO frontline healthcare workers. As part of the CDC's *Project Firstline*, NCUIH was chosen as a partner in a national collaborative. The first task was to conduct listening sessions with frontline UIO staff to learn more about IPC practices in the context of the COVID-19 pandemic. Thirty staff from 16 UIOs, representing full ambulatory, limited ambulatory, outreach and referral, and outpatient and residential treatment programs participated in virtual video focus groups in July of 2020. Thematic and content analysis protocols guided data analysis and coding. Analysis of findings generated four major themes: staff adaptation in the context of resilience; responsibility and duty to protect patients, families, and coworkers; mental and emotional issues for UIO staff; and IPC challenges in the context of COVID-19. Participants' challenges ranged from lack of access to personal protective equipment (PPE) to the absence of standardized training. Significant disparities in social determinants of health experienced by Native American and non-Native populations served by UIOs create additional challenges to the delivery of and access to care during the pandemic. The diverse array of tribal cultural values and contexts of the people and communities served by UIOs reportedly serve as both facilitators and barriers to care, awareness, and uptake of infectious disease public health practices.

## Introduction

Severe acute respiratory syndrome coronavirus 2 (SARS-CoV-2), the virus that causes Coronavirus Disease 2019 (COVID-19), has created significant challenges for outpatient health care providers and patients across the United States (U.S.) (Corallo and Tolbert, 2020; Provenzano et al., 2020; Slavitt, 2020). Healthcare providers have had to quickly shift clinical practice and systems of care in response to the pandemic. Urban Indian Organizations (UIOs) are no exception. UIOs provide a broad array of health and public health services for American Indian and Alaska Native (AI/AN) populations and other underinsured and uninsured populations in 42 major urban areas across the U.S. (National Council of Urban Indian Health, 2020).

There is scarce research about the risk, severity, outcomes, or protective factors for COVID-19 inclusive of Native American populations in the U.S. However, sporadic, slowly emerging data indicate that COVID-19 disproportionately impacts Native people and other people of color (Arrazola et al., 2020; Dorn et al., 2020; Hatcher et al., 2020; Laurencin and McClinton, 2020; Tai et al., 2020; Kakol et al., 2021). A U.S. Centers for Disease Control and Prevention (CDC) study found COVID-19 case rates for American Indian and Alaska Native people were 3.5 times higher than that of non-Hispanic white persons (Hatcher et al., 2020). A more recent study found the overall age-adjusted mortality rate for American Indian and Alaska Native people was 1.8 times higher than that of white persons; moreover, American Indian and Alaska Natives aged 30-39 were 11.6 times more likely to die than their white counterparts (Arrazola et al., 2020). Kakol et al. (2021) and Tai et al. (2020) argue that because of pre-existing health disparities linked to social determinants of health, Native peoples and other people of color are at a higher risk for COVID-19 infection. Underlying conditions such as diabetes and heart disease create persistent gaps in health status and increase the risk of complications from COVID-19 (Kakol et al., 2021). Further, long-standing historical, structural, and social injustices continue to perpetuate these inequities in social determinants of health (Rodriguez-Lonebear et al., 2020; Tai et al., 2020). Due to limited resources, including a lack of personal protective equipment (PPE), hospital beds, and chronic funding shortfalls, the Indian Health Service's (IHS's) capacity to serve the rapidly increasing number of cases of COVID-19 in tribal communities is limited (Dorn et al., 2020). Lack of treatment options and the inability to mitigate community transmission affect mortality rates across the country for Native peoples (Dorn et al., 2020; Kakol et al., 2021).

### Urban Indian Health Care

According to the U.S. Census Bureau (2010), approximately 70% of American Indian and Alaska Native people live in urban areas (2010), with 25% living in areas served by UIOs (Indian Health Service, 2018). U.S. legislative and executive branches have long recognized that the federal government's treaty responsibility to provide for the health of American Indian and Alaska Native people is not restricted to the borders of reservations. The Federal Trust Responsibility instead includes the provision of healthcare wherever they reside (National Council of Urban Indian Health, 2020). The Indian Health Care Improvement Act of 1976 (1976) (IHCIA) defines urban centers as “any community which has a sufficient urban Indian population with unmet health needs to warrant assistance under subchapter IV, as determined by the Secretary.” As independent non-profit health facilities, each UIO defines its service population, which can include both American Indian and Alaska Native and non-Native patients (National Council of Urban Indian Health, 2020). Over half of UIOs are Federally Qualified Health Centers (FQHCs), 11 of whom receive funding from the Health Resources and Services Administration, four also receive Healthcare for the Homeless program funds (National Council of Urban Indian Health, 2020). In 2018, UIOs reported serving 179,196 clients, of which 72,243 were American Indian or Alaska Native, according to IHS data (National Council of Urban Indian Health, 2020).

The IHS has been the primary source of funding for American Indian and Alaska Native healthcare as part of the Indian Health Service/Tribal Health Programs/Urban Indian Organization or I/T/U system of care (Indian Health Service, 2020). UIOs are principally funded by a single line-item in the IHS budget that constitutes <1% of the total IHS budget with a directive to serve members of all 574 federally recognized tribes and urban Indians (Indian Country COVID-19 Response Update, 2020). As a result of decades of chronic underfunding, UIOs are left to rely on a patchwork of third-party reimbursement options and grants to maintain operational and financial viability (National Council of Urban Indian Health, 2020).

The COVID-19 pandemic has exacerbated existing operational issues for some UIOs and created new problems for others (Indian Country COVID-19 Response Update, 2020). According to a survey conducted in September of 2020, 60% of UIOs reported a reduction in operating hours due to COVID-19. Forty percent of respondents are unable to provide testing, and an additional 20% of UIOs are unable to meet testing demand citing inadequate PPE or testing supplies, staffing issues, the rising cost of supplies, limited local or state support, or other challenges. No single UIO respondent felt they had the resources to conduct contact tracing, and none felt there were sufficient public health community resources to conduct contact tracing as needed for their patients. Nearly seventy percent of UIO respondents indicated their facilities lacked adequate space for triage or isolation. Every UIO respondent indicated a need for health information technology staff to facilitate telehealth service demand. Many UIOs requested funding and support for a wide range of telehealth service issues and equipment, including funding to assist low-income patients who lack the technology or the capacity to access telehealth services for virtual appointments. More than half of UIO respondents indicated they would be unable to maintain operations for more than 30 days if any lapse in federal funding were to occur, such as happened during the 2018–2019 federal government shutdown, which resulted in three UIOs permanently closing (Indian Country COVID-19 Response Update, 2020).

### U.S. Indigenous Population Terminology

In the U.S., American Indian and Alaska Native “…race is a political status that confers access to health care services under treaty obligations of the U.S. government” for federally recognized tribal members (Hatcher et al., 2020, p. 1168). In response to this political designation by the U.S. government, American Indian and Alaska Native (AI/AN) terminology has been largely adopted in the research literature. More recently, the term Native American has been used with increasing frequency, and the terms AI/AN and Native American are often used interchangeably (Connolly et al., 2019). The National Congress of American Indians (National Congress of American Indians, 2020) defines Native Americans as inclusive of all Native people from the U.S. and its territories, including Alaska Natives, as well as Native people from Canada, Mexico, Central and South America who are residents of the U.S. Listening session participants most often used the terms “Indian” or “Native” in their discussions or references to their patients or community members. (There is no UIO in the state of Alaska.) In recognition of the title for this special collection, in accordance with the large body of literature surrounding Indigeneity and Indigenous research methodologies, and reflecting the words of UIO participants, the terms Native or Native American will be used throughout the remainder of the publication. References to data, published works, or citations that specifically refer to political or federal designations or references to these populations in some explicit or directed manner, e.g., AI/AN, Alaska Native, American Indian, or by designated tribal affiliation(s), will maintain the original designation when deemed essential.

### Infection Prevention and Control, Healthcare Workers Serving Native Peoples, and COVID-19

A literature review on infection prevention and control (IPC), healthcare workers, Native Americans, and the COVID-19 pandemic identified little U.S.-based research. There is a larger body of available research on IPC and healthcare workers from outside of the U.S. However, the potential relevance to UIOs and Native populations and Native-serving healthcare providers is limited. Therefore, to frame the present article in terms of utility and relevance to the current project and UIO audience, the existing literature review focuses primarily on U.S.-based research or recent systematic reviews or meta-analyses.

In September of 2020, there was minimal research or other published works available in medical or allied healthcare journals focused on U.S. healthcare workers in I/T/U settings during the pandemic. Four identified non-research articles offered “perspectives” from the field, “notes,” or insights into current practices (Close and Stone, 2020; Egan and Bonar, 2020; Kovich, 2020; Rosenthal et al., 2020). Three of these articles described healthcare workers' experiences serving Navajo Nation patients and communities (Egan and Bonar, 2020; Kovich, 2020; Rosenthal et al., 2020), and one described a rural IHS Arizona hospital (Close and Stone, 2020). Three articles described work in hospital-based settings (Egan and Bonar, 2020; Kovich, 2020), including a critical access hospital's expanded public health role that included contact tracing as a means for mitigating the spread of infection in crowded home environments, identifying early cases, and preventing hospital admissions (Close and Stone, 2020). Rosenthal et al. (2020) discussed the critical and evolving role of community health representatives (CHRs) in the Navajo Nation's response to the pandemic. Navajo CHRs have been responsible for an ever-expanding scope of practice, including contact tracing, monitoring curfews, delivering food boxes, and have increasingly gained community trust. Informal themes identified across two or more articles included:

- Adaptation of staff roles
- Adaptation of how or where services are provided, e.g., testing in patient homes, primary care in parking lots, telehealth services
- Importance of and transition to community-based and home-based care and services
- Long hours and high level of intensity of the work for staff
- High prevalence of multi-generational households and high occupancy households, which are high-risk environments for virus transmission
- Importance of partnerships and collaborations
- Need to organize and provide basic supplies for community members, including food and water
- Multiple provider types as options for contact tracing
- Healthcare workers as heroes

No IPC-related research inclusive of healthcare workers in I/T/U settings was identified in peer-reviewed journals when the present article was submitted. Houghton et al. (2020) conducted a rapid review at the start of the COVID-19 pandemic of barriers and facilitators to IPC practices among healthcare workers (not specific to I/T/U settings) during outbreaks. The review categorized findings from qualitative and mixed-methods research. They identified three main categories: (1) organizational factors, including safety climate and communication; (2) environmental factors, including facility concerns and availability and use of PPE; and (3) individual factors, including knowledge, attitudes, and beliefs. The authors outline continually changing guidelines, IPC workload increases, fatigue, a lack of training standardization, and lack of resources, including PPE, as barriers for healthcare workers to perform optimally (Houghton et al., 2020).

There are significant gaps in the research literature about the impact of COVID-19 on Native-serving healthcare providers, whom the authors conjecture face some of the most significant challenges given the populations served and systems in which they operate. Furthermore, there are implications for the unique cultural and historical aspects of Native populations regarding disease responsiveness (Santosham et al., 2007). The latter has implications for healthcare workers that are as yet unexplored in the context of COVID-19. For decades, studies of infectious disease epidemiology, prevention, and treatment among Native populations have provided insight into interventions and care that have reduced global morbidity and mortality (Santosham et al., 2007). The UIO listening sessions' original aim was to gather information to inform the training needs and preferences of UIO healthcare staff. However, the systematic qualitative exploration and analysis of overall IPC perceptions and experiences amid the COVID-19 pandemic in this unique segment of the healthcare workforce that serves a population consistently under-represented in the research literature is the first of its kind. This paper provides a rare glimpse into issues for the UIO workforce, and the findings may lead to greater understanding and serve as a foundation for future research.

## Methods and Materials

### Aims

NCUIH is the only national non-profit organization devoted to supporting and developing quality, accessible, and culturally competent health and public health services for American Indians and Alaska Natives living in urban areas. NCUIH represents the 41 Title V UIOs funded by and under contract with the IHS. The CDC provided funding to NCUIH via a cooperative agreement to participate in *Project Firstline*, a national training collaborative for healthcare infection prevention and control training. *Project Firstline* aims to provide foundational and practical knowledge directly to more than 6 million frontline healthcare personnel and targeted components of the public health workforce to protect the nation from infectious disease threats, such as COVID-19.

Two of the first partner activities undertaken included a national healthcare worker survey and listening sessions facilitated by national collaborative partners. The national survey, developed and hosted by the CDC, was distributed electronically by NCUIH and other national partners. Survey questions solicited feedback on training preferences, including possible delivery mediums, topics, formats, and timing. Survey data was only reported to partners in aggregate form and was not made available for separate analysis. Results of the survey are not the focus of this paper. Locations and other identifiers have been omitted from quotes taken from survey responses.

Listening sessions with frontline healthcare personnel and other relevant stakeholders were convened individually by NCUIH and other external partners at the CDC's request. The primary objectives of NCUIH's listening sessions were to understand the needs, challenges, and opportunities for improvement in IPC training and systems from the perspective of UIO frontline healthcare workers in the context of the COVID-19 pandemic.

### Design

*Project Firstline* listening sessions were not intended as research. At NCUIH's discretion, qualitative research methodologies that included Thematic Analysis protocols (Braun and Clarke, 2006) guided the process, along with other qualitative methods that were implemented to ensure analytic rigor, reliability, and validity of the findings (Morse, 2015; Hennink et al., 2019).

### Setting and Participant Recruitment

IHS funds four types of UIO programs: full ambulatory, limited ambulatory, outreach and referral, and mental and substance abuse treatment centers (outpatient and residential). At the time of this publication, 22 full ambulatory programs provide direct medical care for 40 or more hours(h) per week. Six limited ambulatory UIO programs provide direct medical care for <40-h per week. Ambulatory programs offer diverse services and medical care, including nutrition, oral health, behavioral health, substance abuse, elder services, and more. Five outreach and referral programs do not provide direct medical services but instead provide referrals to specialists and offer community programs, wellness, and prevention services. Eight programs offer a spectrum of services focused on residential or outpatient behavioral health services, including substance abuse. Staffing levels, patient encounters, service delivery models, and program expectations differ between UIO programs (National Council of Urban Indian Health, 2020).

UIO staff were recruited for the virtual listening sessions primarily through convenience and snowball sampling, with later purposive sampling to ensure participation from each of the four UIO program types (full ambulatory, limited ambulatory, outreach and referral, and treatment centers). Emails were sent to the NCUIH UIO e-news list over several weeks (~1,918 recipients; 4 emails). Also, targeted emails were sent to specific UIO Chief Executive Officers to request a recommendation for a staff member to participate in upcoming sessions in order to increase representation for certain UIO program types. Incentives for participation in the information gathering process, which included taking a modified CDC pre-session survey, participation in the two-hour listening session, and providing feedback on a summary of findings (member checking), were offered ($150 gift card).

### Data Collection Procedures

Five 2-h virtual video listening sessions (focus groups) were convened over two-weeks in July of 2020. Three sessions were scheduled for a maximum of up to 10 participants. Additional sessions were added to accommodate alternate days of the week for clinical providers (by request), schedule new participants from previously un-represented UIO program types, reschedule sessions for participants who missed original sessions, and triangulate initial themes identified in earlier sessions. Half of the NCUIH *Project Firstline* staff were tribally enrolled, including the primary facilitator and moderator. Listening session questions were derived from a CDC-developed semi-structured interview guide (see Supplementary Materials) adapted to allow for more culturally relevant and UIO specific discussion with participants. The interview guide was modified before and between sessions to facilitate conversational flow, incorporate new prompts focused on emerging issues, and ensure feedback was obtained on all critical areas. Online polling software was incorporated throughout the sessions to ease participant comfort in providing feedback in a group setting and facilitating sharing for limited audio or video capacity. An online survey distributed after each session solicited recommendations for process improvements and provided an opportunity for additional comments. Staff reviewed survey results immediately and incorporated relevant suggestions in future sessions. Video sessions were recorded, an edited transcription and set of notes were created, and then online recordings were deleted.

### Analysis

Multiple strategies were incorporated to enhance rigor in the analysis process (Morse, 2015). Data collection and analysis occurred concurrently. After each session, a peer (team) debrief was convened, and a list was generated that consisted of significant, new, or emerging themes. During subsequent peer debriefs, staff reflected on persistent themes as well as identified new or emerging themes. Persistent and potential themes identified during early peer debriefs were cross-checked or clarified during future listening sessions with participants to triangulate themes from earlier sessions (Morse, 2015). Braun and Clarke (2006) argue that saturation, a commonly used term in qualitative research to imply analytic rigor vis a vie information redundancy, is not a thematic analysis tenant. However, it was apparent that saturation was achieved on several significant themes identified via peer debriefs, confirmed via triangulation with subsequent listening session participants, or that arose naturally in the course of the discussions before coding commenced (Hennink et al., 2019). Dedoose Version 8.0.35 software (Los Angeles, CA: Socio Cultural Research Consultants, LLC) was used to organize listening session data (edited transcripts and notes), which was then analyzed and coded to indicate further areas of inquiry with feedback on parent and child codes by two reviewers (N.C. and J.C.) from the NCUIH *Project Firstline* team. One reviewer (J.C.) coded certain aspects of data for content analysis (aimed at categorizing responses), and a second reviewer (N.C.) coded primarily for themes. Two reviewers confirmed codes and subsequent themes during multiple encounters until consensus was achieved to ensure credibility and accuracy. Finally, after coding was complete, member checking (Morse, 2015) was conducted to solicit feedback on themes and subthemes via email and through a scheduled follow-up teleconference session.

### Ethical Review

The project was reviewed by the CDC's Human Subjects Advisor and determined to be non-research as defined in 45 CFR 46.102(l); thus, Institutional Review Board review or exemption approval was not required.

## Results

Thirty staff from 16 UIOs and one urban behavioral health office program participated, representing full ambulatory (eleven participants), limited ambulatory (four participants), outreach and referral (eight participants), and outpatient and residential treatment (one participant) programs. Six participants did not identify a program type. A variety of roles and professions were represented among participants (Table 1). Participants also represented a wide range of geographic regions and urban areas.

**Table 1:** Professional role of listening session participants.

**Professional Role**	**Number of Participants**
Registered Nurse / Licensed Vocational Nurse	8
Office Staff / Billing	5
Nurse Practitioner	3
Medical or Nursing Assistant	3
Public Health Professional	3
Behavioral Health / Social Work	3
Clinic Administrator	2
Physician	1
Community Health Worker	1
Dental Assistant	1

Questions in the original interview guide covered four main topic areas. Interview guide topics were (1) Perceptions of IPC (2) Experience with IPC during COVID-19 (3) Training sources and delivery, and (4) Other supports.

### Content Analysis

Multiple questions in the interview guide elicited close-ended or categorical responses. Content analysis of these questions included developing an initial set of code categories by reviewers, followed by assignment, and then comparing code frequencies for respondents across all five listening sessions. The following (Table 2) summarizes content analysis for key questions, with the most frequently identified codes listed in rank order and bold items representing codes selected with the greatest relative frequency within any one category. An asterisk (^*^) denotes if a concept or code was mentioned in all five listening sessions.

**Table 2:** Most frequently identified codes listed in rank order and bold items representing codes selected with the greatest relative frequency within any one category.

**What is your motivation to engage in infection prevention and control practices?**
Protection of patients*
**Protection of family**
Protection of coworkers
Protection of self*
**What are the most critical needs during COVID-19?**
**Space**
**Transportation**
Cleaning, environmental maintenance
Staffing
Training and education
**What is the single most important topic that you think should be addressed in training?**
**Mental health (staff)**
**Staffing**
Patient resources for positive test results*
**Proper use of PPE (ongoing)**
Basics
Cleaning and environment
**Who are your trusted sources of information?**
CDC*
Department of Health*
Staff within UIO*
Other clinics and healthcare providers

An asterisk (*) denotes a term or code that was identified in all five listening sessions.

### Thematic Analysis

Over the five listening sessions, analysis across all participant responses generated four themes. The themes included staff adaptation in the context of resilience; responsibility and duty to protect patients, families, and coworkers; mental and emotional issues for UIO staff; and challenges in the context of COVID-19, which included six subthemes (Table 3). Session facilitators also pointedly explored the unique cultural aspects of working for UIOs. The following listening session participant excerpts included verbatim transcriptions (direct quotes in italics) and staff notes (summary of the discussion not in italics).

**Table 3:** Listening session themes and COVID-19 challenges.

**Majors themes**	**Subthemes of COVID-19 challenges**
Adaptation Responsibility Staff Mental Health Challenges in the Context of COVID-19	Personal Protective Equipment Information Dissemination Space and Service Changes Staffing Issues Lack of Standardized Training Client Social Determinants of Health

#### Adaptation

**Adaptation** was a central recurring theme and appeared in multiple contexts. Most common was the need to adapt to new policies and procedures.

“*…we had standard protocols for the way we handle infection prevention and control, but definitely had to revamp everything and make sure they were up to standards and change how we were going to do things moving forward.”*

*Listening session 2 participant*

Adaptation of roles and responsibilities was a second common, occurring subtheme under adaptation. In concert with staff having either new positions or roles in their facility, e.g., front desk workers or back of house staff conducting temperature checks (“all hands on deck”).

Everyone's role had changed due to COVID-19… As a psychiatric social worker, her role, aside from providing mental health services, now involves psychoeducation related to safety, infection prevention, and general physical health monitoring.

-Listening session 5 participant

Before COVID-19, she did not have a role in IPC. Since the pandemic, she is now responsible for monitoring the temperature of her patients, asking them to use PPE as well as using PPE herself during all her face-to-face sessions. She predominately provides telehealth services, whereas before, “that was not a thing” at her facility.

-Listening session 5 participant

Significant adaptation to facility layout and workflow was common across all operating organizations. Most facilities are continuing to adapt their environment and physical space to be responsive to new policies such as social distancing or conditions like weather (e.g., triaging patients in tents in >100°F outdoor temperatures).

[omitted] indicated that they were having challenges with that on multiple levels… Recently his facility had a quarantine and isolation assessment team from [omitted] assess their ventilation system, which identified that their current circulation would need to be upgraded. They do not have a dedicated room to minimize exposure and will need to put a trailer outside. They are finding roadblocks with space issues. “We are willing to do the training and get certified and put together the layered approach to following up, but at the same time, we are finding roadblocks with space issues.” They moved to telehealth and had staff work from home and had to accommodate that. “We are resilient” “We are trying to negotiate the space challenge.”

-Listening session 2 participant

Nearly all facilities have instituted new workflow processes, new meetings, or more training on various subjects, and there was a reference to the increasingly blurred lines between home and work interactions. The rise in telehealth visits as an option for continuing patient encounters was generally praised, though they present implications for both patients and providers.

Participants indicated in virtually all sessions that these necessary adaptations presented multiple opportunities for improvement. The speed with which changes are implemented (e.g., too fast, too slow), the number of implemented changes occurring at one time, and the need for basic training on new policies or procedures were suggestions participants believe leadership should consider for enhancing future process changes.

Notably, participants often framed the *need* to adapt in the context of the concept of resilience. Resiliency for several participants seemed to take on the meaning of having the capacity to adapt and excel quickly, coupled with the ability to frame or re-frame their situation positively, particularly in challenging or demanding conditions. Many participants feel there is an overemphasis on negative framing in Native healthcare contexts and that their adaptations during the pandemic should be viewed through a lens of positive action. Discussions of resilience and positive re-framing frequently coincided with discussions of the use and maintenance of cultural practices, specifically in Traditional or Native healing contexts and the use of healers for their staff within their organizations.

#### Responsibility

The second most frequent theme was **responsibility**, often discussed in concert with the concept of adaptation. Participants felt that they had a responsibility to their patients (first), families, and coworkers to be knowledgeable and implement safe practices. One participant said,

“*It's necessary for all staff to be trained and to be aware of IPC. We are responsible for keeping our patients and our community members safe.”*

-*Listening session 5 participant*

“*My motivating factor is to prevent the spread of infection. At [omitted], each person is responsible for taking our own temperatures, using hand sanitizer, and wearing masks. Our staff is staggered 50:50 where some staff are on site for two days and then alternate on Fridays while keeping 6 feet distance.”*

-*Listening session 4 participant*

Nearly all participants voiced that everyone has a responsibility or a duty to have at least some broad understanding of IPC. One participant mentioned that they had a sense of ethical duty to care for others' well-being and that engaging in proper IPC was an extension of their ethical duty.

Participants frequently mentioned duty and responsibility related to their motivation to continue training and seek out updates on continually changing protocols and guidelines. Some participants saw a desire for standardized IPC training and standardized information on policies, procedures, or protocols as a means to help increase confidence in their work.

“*However, there are moments when a little bit of fear creeps in and adds to the motivation. Fear of infection and seeing the numbers spiking is a reminder that this is not over yet.”*

-*Listening session 5 participant*

#### Staff Mental Health

Participants discussed an increase in observed and potential **mental health concerns for staff** throughout their facilities, as well as for themselves. This was the third central theme identified across sessions. Fear, fatigue, stress, stigma, and other psychological and mental health issues presented as sometimes subtle and other times not so subtle ever-increasing challenges for participants in the listening sessions. Fear of the potential for family members becoming sick, fear of becoming sick themselves, and worry about at-risk community members were mentioned repeatedly.

“*I don't deal with the frontline. They do the COVID testing… I'll look outside the window. Seeing the fear and the body language…”*

-*Listening session 2 participant*

Several participants mentioned specific negative emotionally-laden scenarios that motivated them to take IPC precautions and maintain community safety. Staff fear of infection mingled at times with their frustration with patients' or community members' lack of compliance with policies (e.g., mask-wearing). Participants suggested this lack of compliance may result from overt disregard for policies, lack of knowledge, or misinformation. Staff frustration also resulted from a lack of consistent messaging from agencies providing COVID-19 guidelines or continuously changing external guidelines, which lead to ongoing changes in internal policies and procedures. Several participants pointedly identified “mental health” as the most crucial topic to address in IPC training.

The Thursday ‘check-ins' were more of a check-in on everyone mentally and how they were doing and how this would affect their family… “They were very emotional, broke down, they didn't know how to respond, they didn't know it was okay to come to the circle, to a safe space and say I need help.”

-Listening session 2 participant

The culmination of by-then several months of rapidly changing policies, procedures, and working under emotionally demanding conditions appeared to be taking their toll on UIO staff.

IPC training was vital to ensuring that staff was informed and supported on an ongoing basis. For [omitted], they use staff meetings, both functional and discussion on stand-ups. Initially, the staff was somewhat hesitant but readily engaged. As the COVID pandemic continued, the staff seemed less open and receptive due to fatigue, secondary trauma, peripheral care required for community and family, and the resurgence in their area. They are trying to do self-care, but this has transitioned into “fatigue,” affecting the donning and doffing practices in the clinic.

-Listening session 5 participant

Some UIOs have also identified an increased demand for substance use and counseling services or other mental or behavioral health needs of their patients, with rising acuity levels, which has translated into increased stress and staff workload.

#### Challenges

Barriers and challenges to infection prevention and control during COVID-19 were specific questions. In addition to analyzing question-specific responses, reviewers examined and coded responses across listening session questions for text excerpts that could be characterized and coded as challenges.

**Personal protective equipment (PPE)** was the most frequently cited challenge and came up multiple times in every listening session. Lack of or limited access to PPE and supplies, concerns about the lack of training about safely putting on (donning) and taking off (doffing) PPE, and staff resistance to wearing masks in the clinic were primary concerns. Though select participants offered examples of clinics that felt they had adequate PPE supplies at the time, several noted they were operating at a very limited capacity. Other examples of promising or successful practices were described. One UIO identified safety teams used to monitor staff for proper donning and doffing procedures, and another described mock drills implemented to prepare staff for procedural changes.

An additional challenge was **information communication and dissemination**. This topic also came up multiple times in every listening session in multiple contexts. For example, the capacity to stay on top of and evaluate multiple external sources' guidance was an ongoing issue.

[omitted] felt that local, state, and federal guidelines also acted as a barrier to implementing IPC. “There is conflicting information. For example, the governor may state that masks should be worn, and then there are other guidance that do not mandate this.”

-Listening session 4 participant

Communication issues also occurred within UIO organizations.

“*Things were changing every day, and sometimes we were out of the loop and doing it wrong. Sometimes new things were implemented, and we were not informed. Sometimes people were just doing things on their own.”*

-*Listening session 2 participant*

These communications and information dissemination challenges also extended out from the UIO. Though UIO staff consistently identified themselves as a trusted source of information in their community in their capacity to educate and reach community members.

In [their] local area, there were 90 different tribes, and it was hard to get everyone on the same page. Their challenge is to get information out, not only healthcare-related but also about available tribal provided services.

-Listening session 2 participant

“*People are reading a lot of stuff. A lot of it on social media. A lot of it is not true. Providing IPC training for our community from the facility would be great training for our Native community.”*

-*Listening session 2 participant*

**Space and facility services** came up multiple times in every listening session. Most UIOs were still not operating at their pre-COVID-19 capacity as of July 2020. Participants noted that the shift to telehealth for many facilities had been a challenge. One participant shared that their dental services had shut down completely, while two others noted that they had to discontinue all patient transportation services. The need to enhance social distancing and reduce patient-to-patient and patient-to-staff contacts has resulted in substantial patient flow and management modifications.

They staggered their visits as a safety measure and even provided curbside pick-up of drugs for their pharmacy. They also provide drive-through COVID-19 testing under a tent.

-Listening session 4 participant

There were many instances in which participants voiced concerns over the physical conditions and small space at their facilities. One UIO had separated symptomatic and non-symptomatic individuals, but due to a lack of available space, had set up a tent outside.

The heat index in [omitted] the day prior was 110 degrees. This is affecting outdoor testing and triage. The facility has obtained tents with air conditioning to help with outdoor screening/testing for COVID-19 patients. Wearing PPE in the heat is a safety issue.

-Listening session 1 participant

Social distancing requirements and facility capacity issues have contributed to limitations in the number of medical staff allowed on-site at any one time in some facilities. **Staffing issues** also came up multiple times in every listening session.

“*We are kind of a small facility, so we really only had two janitorial staff but had increasing needs for more thorough and intensive cleaning. Along with this, it takes time to hire additional clinical staff. We want to hire additional trained nurses and support staff to help meet increased needs. We are going to have to take more time with each patient. Increased needs for testing. Our biggest hurdle is ramping up on staffing.”*

-*Listening session 2 participant*

Staff contact with confirmed or suspected COVID-19 positive persons at work and in their personal lives was an issue, especially for facilities with already small staff sizes.

“*Staff members who travel out of state and that puts them at high risk for exposure.”*

-*Listening session 2 participant*

Still, other staff who have chosen to leave the workforce, not return out of fear or concern for themselves or family members, or were not allowed to return because of potential risk cause additional strain on the UIO workforce.

Some staff were considered essential but were scared and wanted to remain home. Some staff telecommuted, some stayed home, and those considered high-risk were sent home.

-Listening session 4 participant

**Lack of standardization of training**, concerns about ongoing compliance by some individual staff members, and rapidly changing protocols or procedures were consistent themes identified across all listening sessions.

“*We definitely do run into problems with people being resistant to change, not wanting to make a change, change for a while, and then revert back to the way things always were. That's our biggest challenge.”*

-*Listening session 2 participant*

Several examples of UIOs that had developed proactive, responsive, and robust systems for training and monitoring IPC procedures were shared. While 74% of listening session respondents who responded indicated their UIO currently provided some type of IPC training, available training sources and program requirements varied widely. Broadly, there was a desire for more training, including more staff (not just clinical), that was timely and included ongoing follow-up for compliance.

This [standardized training] was not required. There was no standardized, formal introduction and training; regarding what PPE looks like and the benefits, structure, and process. She hopes that they can have formal training.

-Listening session 5 participant

Finally, participants in multiple sessions identified a range of **social determinants of health** that they believe disproportionately impact the Native clients seen at their UIO. These issues, which predated COVID-19, have further exacerbated challenges for providing care and services during the pandemic. According to the CDC, “social determinants of health are conditions in the places where people live, learn, work, and play that affect a wide range of health and quality-of-life risks and outcomes” Centers for Disease Control Prevention (2020). Participants most frequently discussed the lack of insurance or underinsurance. Additional social determinants of health identified by participants included

- Lack of internet access hampers the ability for patients to use telehealth services
- Disruptions in the clinic and public transportation systems for patients who have no other transportation alternatives
- Lack of running water or electricity for sanitation
- No access to masks or ability to clean reusable masks
- Community resources shut down due to COVID-19 or limited service hours
- Increased number of homeless and those facing food insecurity
- Lack of access to basic hygiene supplies like hand soap.

Two participants noted a shift in UIO patient population demographics. One cited an increase in the number of homeless patients seeking services, and the second noted an increase in the number of formerly incarcerated patients. Furthermore, both respondents said those clients had no other option for formal health services at that time beyond the UIO.

#### Cultural Aspects of UIO Work

Most UIOs see clients of all races and ethnicities. UIO Native patients come from many different tribes and represent various cultural practices and customs. One participant noted his work with 90 different tribes from their UIO service area. Participants shared examples of UIO cultural services that have included Sun Dances, sweat lodges, sage and sweetgrass for smudging, traditional healers, traditional medicine, and cultural leaders who lead prayers for both staff and patients. Culturally appropriate services bring value to both the communities served and staff who themselves may be Native American.

“*It's kind of hard to explain. I've always lived in two different worlds. My cultural side was at home, with my family. Work was work. So, I turned that side off. Here [at the UIO] everything is put together. I'm learning how to bring out the one side now, and I don't have to shut part of myself off.”*

-*Listening session 2 participant*

However, COVID-19 has dramatically disrupted both clinical and cultural practices for UIOs and the communities they serve. Additionally, participants note there is some skepticism about the disease among community members.

“*They are strong spiritual people who believe this COVID-19 is not natural.…There has been much stubbornness where some people feel like they are immune.”*

-*Listening session 4 participant*

Within the urban setting, traditional practices are still occurring, and funerals still occur, but they [UIOs] are trying to implement prevention measures. As it relates to the sweat lodge ceremony, they are trying to put precautions out there. While in the clinic, it becomes challenging when the response is, “…not our disease”.

-Listening session 4 participant

A follow-up discussion on preliminary findings (part of member checking) included several UIO staff from the original listening sessions from different parts of the country. These participants concluded that what was identified as ‘skepticism' in preliminary coding and quotes is sometimes manifested as overt “denial” by some community members. One participant reflected on an elderly Native patient who personally experienced severe illness due to COVID-19, yet commented that it was not their [Indigenous people's] disease. The group postulated that skepticism or denial was potentially a root cause of behaviors that put others at risk, contributing to patient resistance to practices such as wearing masks, social distancing, or foregoing cultural ceremonies to protect themselves or their families. Native perceptions of infectious diseases (e.g., smallpox or AIDS), chronic diseases such as diabetes, and even cancer as “white man's” diseases have been discussed in research and health literature (Jones, 2006; McLaughlin, 2010; DesJarlait, 2017; Pfeiffer and Gilley, 2017). The origins of these diseases relative to the white man have been described as punishment for sin or immorality with roots in religion, human-made biologics, disruption of traditional Native ways caused by adopting the white man's lifestyle, or conspiracy by the government to perpetrate genocide. In virtually all cases, these perceptions and beliefs are often complex, deep-seated, and are an obstacle to care and treatment until overcome (Jones, 2006; McLaughlin, 2010; DesJarlait, 2017; Pfeiffer and Gilley, 2017).

Trust is a critical issue in Native communities. Although UIO healthcare staff generally referenced the CDC as a trusted source of information (65% of listening session participants who responded agreed), they indicated that their community members would be far less likely to trust the CDC. However, participants stated that their community members trusted UIO staff and ultimately would trust UIO guidance even if it originated from the CDC. There was also consensus that (1) broad community outreach and awareness on infection prevention and control training (2) through trusted sources (such as UIOs) to Native people living in urban communities (3) done in a culturally competent manner, and (4) accompanied by needed resources was a high priority. Participants provided several examples of culturally centered practices being explored and employed at their UIOs since the start of the pandemic.

In their staff meeting, her staff members discuss the cultural medicines that could be useful along with current practices. She also shared that on-site was a cultural person who shared this information. Her center also provides an area with sage and sweetgrass so that people can smudge themselves (if they choose) when they come in for appointments.

-Listening session 4 participant

## Discussion

The COVID-19 pandemic has at times overwhelmed virtually every component of the U.S. health system, resulting in shortages of staff, supplies, and infrastructure, as well as rationing and triage of care (McGuire et al., 2020), and UIOs are no exception. Native people served by UIOs and other tribal health providers suffer disproportionate rates of infection owing to a backdrop of significant mental health and health disparities, disparities in social determinants of health, and significant structural inequities (Dorn et al., 2020; Hatcher et al., 2020; Tai et al., 2020; Kakol et al., 2021). UIO listening session participants provided novel insights into staff perceptions and experiences directly from the frontlines of care five months after the first COVID-19 case was diagnosed in the U.S. Though conversations were framed in the context of IPC practices and programs and designed to inform related training resources and protocols, the findings have relevance beyond training needs. At the same time, they preserve UIO healthcare workers' experiences at a unique moment in history. The four major themes generated from discussions included **adaptation** in the context of resilience, **responsibility** and duty to protect, **mental** and **emotional** issues for UIO staff, and **challenges** in the context of COVID-19. Multiple challenges were identified by participants, many that are perhaps not unique to the UIO setting. Significant disparities in **social determinants of health** create a unique, stark divide for the Native and non-Native populations served by UIOs relative to other mainstream healthcare providers' patient populations. According to listening session participants, the diverse array of tribal cultural values and traditions of the people and communities served by UIOs serve as facilitators and barriers to care, awareness, and uptake of infectious disease public health practices.

It is vital to consider findings and recommendations within the context of documented historical trauma and losses experienced by Native peoples in the U.S. Genocide, colonization, forced removal, efforts at assimilation via boarding schools or relocation programs, and other recurrent attempts to eliminate traditions and cultures of U.S. indigenous populations have resulted in government and institutional distrust (U.S. Commission on Civil Rights, 2018). Emerging research indicates perceptions of historical trauma and losses have increased mental health sequelae among American Indian people during the COVID-19 pandemic (John-Henderson and Ginty, 2020). Historical trauma and heightened stress mingled with mixed messages from various government entities as a result of continued learning about the virus likely lead to confusion and further foster the sense of distrust among Native communities.

### Comparison With Existing Literature

Many findings identified in UIO listening sessions are reflected in the recent scientific literature. Several themes resonate with results from Liu and colleagues' (Liu et al., 2020) study of hospital-based healthcare provider experiences during the early stages of the COVID-19 crisis in China. A duty to patients and community (responsibility), challenges unique to COVID-19, and resilience amid these challenges emerged as three primary themes from their interviews with 14 nurses and physicians in the Hubei province. Additional commonalities were embedded in the article narrative. Themes that parallel experiences of UIO staff included adaptation in work environments, facilities, workflow, roles, and responsibilities; communication challenges; continually evolving protocols; increased stress, fatigue, grief, and fear of infection for themselves, colleagues, or family; and concerns about adequate PPE, supplies, and staffing. Unique to the Hubei study, their providers identified multiple forms of support that offset their experiences of powerlessness, grief, stress, and physical and mental fatigue that culminated in a phenomenon labeled “transcendence.” Systems of support for the Chinese healthcare workers included hospitals that provided logistical resources (PPE, food, and accommodations), ongoing IPC training and support to enhance feelings of safety and group connectedness, and provision of counseling services, in addition to vast social support networks that included colleagues, family, and community. Healthcare workers employed in the Hubei acute care settings presented with relative advantages compared to UIO staff regarding access to resources. Resource abundance on the part of Chinese workers was a significant divergence in findings from UIO staff experiences. Though mental health issues were also common among UIO listening session participants, there was little discussion of systematic approaches to psychological support or resources to address these concerns.

There is also marked congruence in findings from the UIO listening sessions with Houghton et al. (2020) review of barriers and facilitators to healthcare workers' adherence to IPC guidelines for respiratory infectious diseases. Of twenty studies included in the final sample, only one was U.S.-based. Most sampled studies (15) were situated in hospitals, with two non-U.S. studies based in primary care or family practice settings. Several new themes emerged in the current analysis of UIO experiences relative to the systematic review. These differences may be attributed to the setting (primary care vs. acute care), unique characteristics of the population served by UIOs, or perhaps the sustained impact of the COVID-19 pandemic on the U.S. population. See Table 4 for a comparison of findings, including themes identified among UIO participants not identified in the review. Specific to the theme of mental health issues, even though neither mental health, psychological issues, nor stress was explicitly identified as one of the 26 key findings, narrative excerpts in the Houghton et al. review contained several associated terms including fear, frustration, fatigue, and emotional distress (2020).

**Table 4:** Comparison of UIO listening session findings to systematic review of barriers and facilitators to infection prevention control.

**Findings from Houghton et al. (2020) Review that *Aligned* with UIO Listening Session Findings**	**Findings from Houghton et al. (2020) Review *Not* Identified in UIO Listening Session Findings**	**Findings Unique to UIO Listening Sessions**
Long, ambiguous, or conflicting guidelines created uncertainty	Adherence influenced by the support received from the management team	Mental health issues for UIO staff with possible implications for IPC practices
Frequently changing guidelines caused healthcare workers to feel overwhelmed	Guidelines workers perceived as impractical were hard to implement	The implication of telehealth on clinical and IPC practices
Fatigue and increased workload from IPC practices a barrier to adherence	Multiple methods and platforms for communications useful to ensure staff are kept up to date	Staffing issues (vacancies due to risk, exposures, and increased work demands)
Clear communication and sharing updated information within organizations critical to success	Frontline healthcare workers have difficulty balancing role as IPC trainer with clinical duties	Role of social determinants of health and need for UIO patients
Lack of training on specific infection information and PPE needs contributed topoor implementation	Access to handwashing facilities and surface cleaning supplies identified as key factors in adherence to IPC measures	Implications of culture for the Native population served by UIOs for modifications in traditional practices and medicine, provision of care, and public health practices
Lack of mandatory training and accompanying skills assessments contributed to lack of adherence	Personal knowledge of patient or colleague infection facilitated adherence to IPC guidance	
Healthcare facility with sufficient space to isolate patients viewed as a key to adherence to IPC methods	Workers recognize the responsibility to increase knowledge but must have evidence, rationale, and support to do so	
Adequate ventilation, isolation rooms, anterooms, and shower facilities are required to achieve adequate IPC measures	Workers had to value the importance of IPC to increase adherence and incorporate IPC into routine practice	
Patient/workflow measures, e.g., minimize overcrowding, route control, fast-tracking infected patients, and other measures to reduce risk seen as essential tools to protect staff and patients	Face masks could be perceived as frightening, cause isolation, or stigmatizing for patients and may reduce the use	
Inadequate supplies of appropriate PPE perceived as a serious concern	Discomfort wearing PPE may reduce adherence; ensuring proper fit may help	
Need for PPE increases as outbreaks intensify; requires attention to increasing PPE supply line needs as the outbreak continues		
Knowledge limitations among specific team members can be a barrier to overall ability to adhere to IPC guidelines		
Use of PPE, particularly masks, not always seen as important for healthcare workers		
Fear of infecting self or others facilitated adherence to IPC guidelines		
Workplace culture and colleagues can act as a barrier or a facilitator to adherence		
Healthcare workers felt a duty to care for their patients		

The mental health impact of COVID-19 on healthcare workers is a growing concern across the U.S. and the subject of a rapidly expanding body of scientific literature. A subsequent scan of the literature returned nearly 200 journal articles that primarily originated outside of the U.S. In the aftermath of the 2003 SARS outbreak, DiGiovanni et al. (2004) write that healthcare workers suffered from a range of issues that ultimately affected their mental health such as fear, the inconsistency of measures between information distributors, lack of logistical support, psychological stress, and poor communication to the public. Brooks et al. (2020) identify the psychological impacts of quarantining, including for healthcare staff, who reported deteriorating work performance as a result. Notably, Brooks et al. (2020) identified no strong evidence of any demographic factors linked with poor psychological outcomes after quarantine. Two recent rapid systematic reviews aimed to assess epidemics and pandemics' psychological impact on healthcare workers' mental health (Preti et al., 2020; Stuijfzand et al., 2020). Post-traumatic stress disorder (PTSD), depression, anxiety, burnout, and a host of other psychological symptoms were found to affect healthcare workers up to 3 years after epidemics and pandemics in both reviews. Resilience, perceived organizational support, confidence in protective measures, training, and trust in IPC measures instituted by organizations reduced adverse psychological outcomes.

Conversely, inadequate training, exposure to high-risk environments or perceived personal risk, low organizational or personal social support, and limited coping mechanisms increase the risk of psychological sequelae. A limited selection of evidence-based interventions was identified (Preti et al., 2020; Stuijfzand et al., 2020). Beyond the personal impact of UIO staff endorsements of feelings of fear, fatigue, stress, and other mental health issues, one UIO staff participant shared an anecdote detailing the direct negative impact of long-term mental fatigue on IPC practices, specifically reduced care and attention in donning and doffing PPE. Research identifying a direct causal relationship between fatigue, psychological symptoms, or emotional issues and lapses in IPC was not identified in the literature search. However, there is a substantial body of literature documenting the link between fatigue and risks to personal safety, patient safety, and adverse events (The Joint Commission, 2018). Thus, the hypothetical assumption that UIO staff experiencing these issues are at increased risk of exposure to SARS-CoV-2 due to lapses in practice is plausible.

### Recommendations for Practice, Policy, and Research

#### Practice Recommendations

There are implications from the findings for practice, policy, and research. Given the grounding of this work in frontline UIO healthcare workers' experiences, there are clear and actionable implications for practice that are further supported by recent research. Consistent across the literature are recommendations for providing sufficient access to PPE and protective measures, and enhanced knowledge of IPC and training, with evidence to support the positive short and long term mental health implications of addressing these needs (Houghton et al., 2020; Liu et al., 2020; Preti et al., 2020; Stuijfzand et al., 2020). Houghton et al. (2020) identified additional facilitators that included the importance of how guideline content is communicated, manager support, the culture of the workplace, physical space, duty to provide care, and also noted the need to be inclusive of all facility staff in training (not just healthcare providers). Enhanced education and communication approaches can reduce fear and uncertainty and increase trust by enhancing IPC skills mastery and providing personnel responsible for continuous training, monitoring, and supervision of IPC practices. Organizations can reduce anxiety and other concerns related to virus transmission to family members and staff with training, enhanced safety practices and procedures, supportive conversations, and education about best practices surrounding mitigation and risk reduction for returning home after work (Liu et al., 2020).

Also, the following consolidated set of recommendations derived from the two systematic mental health reviews for supporting healthcare workers during a pandemic is offered for consideration:

- Support and enhance resilience and coping strategies through primary preventive strategies, including consideration for computer-assisted resilience training with interactive, reflective exercises
- Develop and deliver online support services
- Establish a mental health support team for healthcare staff
- Implement widespread staff screenings for perceived safety risks during the outbreak
- For staff with identified concerns, consider multi-phased intervention inclusive of psychological first aid, psychoeducation, and cognitive-behavioral therapy (CBT)
- Managers should foster peer support programs, implement programs to demonstrate organizational support, and routinely provide updated information
- Allow healthcare staff to volunteer for high-risk assignments, rather than impose a requirement
- Implement mental health screenings after the outbreak and at long term intervals.

UIO services and the Native and non-Native populations served are diverse, as are the individual organizations and communities' strengths and weaknesses. Findings from this publication and the literature may provide a framework and starting point for a systematic assessment of a UIO's organizational needs and capacities regarding IPC practices and policies.

#### Policy Recommendations

Policy recommendations are multidimensional. There are both minor policy considerations, meaning organizational rules and practices, and major policy needs, including federal and state policies (Eyler et al., 2016). Within the adaptation theme specifically and throughout discussions, UIO staff acknowledged the importance of organizational policies to their perception of an adequate (or inadequate) response to the unfolding pandemic. UIOs with strong, existing, up-to-date policies and procedures and the infrastructure to rapidly create, review, and deploy policies were better positioned than those who did not have this capacity. Standardized IPC training is needed to support UIOs systematically across the country. As healthcare providers, in a time of scarce resource allocation, UIOs should be prioritized for PPE and other resource allocation at the local, state, and federal levels. UIOs have great diversity in size, scale, and operational capacity. They need comprehensive *support* to update and maintain facilities and operations. Although additional funding to address PPE and other essential supply and resource shortages is an important step, it does not provide a legitimate solution to significant resource shortages, and critical gaps remain regarding healthcare worker safety and training. For instance, funding can allow UIOs to make purchases or new hires. However, when local, regional, or state supply chains are depleted, and resources are being diverted to larger or resource-rich systems or communities, PPE may still be unavailable due to high costs—or it may even be entirely unavailable for purchase through suppliers, or staff may not be willing to work out of concern for personal safety. Principles of health equity, access to national and state stockpiles, and justice and mitigation of health inequities (National Academies of Sciences Engineering Medicine, 2020) in resource allocation strategies (similar to those recommended for vaccines) are needed to facilitate the return to normal or near-normal operations. Federal and state policies must recognize the crucial role of UIOs and other safety net providers, prioritize and address the most at-risk populations' needs.

#### Research Recommendations

There are significant research gaps, which are too often not inclusive of Native populations and community needs. These gaps exist across the spectrum of scientific and medical topics. Infectious disease, epidemic, outbreak, and healthcare workforce-focused literature reviewed for the present article historically and at present primarily originates outside of the U.S. However, within the body of research identified from the U.S., there is a particularly noticeable absence of Native American population representation. The present special collection and future-focused special collections are a significant contribution. It is plausible that the scarcity of publications results from the dearth of research conducted in tribal and urban settings. Unfortunately, case studies and experiences may not be enough to attract and compel policymakers to action. Until research barriers can be identified and removed within these communities and among Native-serving health organizations, creative solutions to fill the void in published literature must be identified. For example, data gathered from systematic assessments and evaluations conducted as part of projects and programs, such as the current project, should be published to serve as a foundation for future research. When research data *is* available, researchers must make the publication of findings a priority. The full spectrum of topics discussed in the present article represents gaps in the research literature. There is limited or no research originating in tribal or urban Native communities on essential topics related to COVID-19, including:

- Infectious diseases, outbreaks, or epidemics involving the I/T/U healthcare workforce
- Effective IPC practices or programs
- Cultural facilitators or barriers to preventive public health practices among Native patients
- Patient and community conceptualization of COVID-19 as a “white man's” disease
- Risk and protective factors for transmission or infection specific to Native American communities
- Role of social determinants of health
- Utility of social vulnerability indices created by the CDC in identification and prioritization of pandemic response efforts.

In fact, each theme or challenge in the present publication reflects an unexplored domain for health research among tribal healthcare workers or Native patients in virtually every health care setting and geographic location they may be served.

## Strengths and Limitations

This paper is, to the authors' knowledge, the first to employ rigorous qualitative methodologies to explore and publish the perceptions and experiences of frontline UIO healthcare workers or any healthcare staff who care for Native American people during the COVID-19 pandemic. A brief structured search of peer-reviewed literature identified no research studies that have assessed outpatient provider (e.g., primary care, family practice, outpatient provider) experiences with COVID-19 in the U.S. at the time of submission. Conversations with UIO staff touched on a diverse array of topics from culture to care patterns. Although these conversations are thought to represent the spectrum of holistic care provided by UIOs, they are seldom discussed in the context of one study or publication. The sessions included participants from multiple disciplines and professions. They all indicated that IPC practices and procedures are now a dominant reality in their work at the UIO. This new reality calls for a richer understanding of the implications of COVID-19 for the entirety of the UIO workforce, which this publication offers. The direct translation of findings to facilitate training, assist with policy development relative to COVID-19, and provide resources inclusive of UIO healthcare workers' needs is a significant and relevant strength. Enhancing the knowledge of a healthcare workforce informed by those on the frontlines during a pandemic has the potential for immediate patient and community benefit.

There were several limitations associated with the approaches employed for the project. As noted, this project was not a research study; instead, the primary aim was to gather information to inform the training and education needs of the UIO staff during the present pandemic. The findings presented here are meant to contextualize understanding specific to the UIO staff experiences with IPC during the COVID-19 pandemic and are not generalizable. Not all UIOs were represented among the volunteer participants. Due to over-encumbered frontline staff at many facilities, there was likely selection bias. Facilities that have a larger workforce may have been more likely to participate in sessions. However, based on the variety of professions, facility types, and facility sizes observed in the listening sessions, this seems unlikely. Limited demographic information for participants was available. Sessions were virtual, and as a result, this may have impacted the ability to build rapport with participants. The semi-structured interview guide, which was not piloted, was designed to elicit specific feedback on training needs and potentially influenced responses (e.g., participants' terminology). While minor, interview guide revisions took several sessions to achieve maximal discussion with the least amount of facilitator intervention. The wording on questions changed slightly over the sessions, which may have led to variation in responses from the first to the last session.

## Conclusions

UIOs, like other healthcare facilities around the country, are facing an unprecedented time in healthcare. UIO workers understand the unique contexts they occupy in terms of health equity, culture, and education. Listening session participants voiced a desire for a standardized IPC training program, policies, and procedures that acknowledge the unique cultural and tribal contexts in which they operate, specific to the I/T/U healthcare system. While identifying training needs was the core purpose of sessions, we were compelled to give voice to issues identified by UIO participants in discussions of their experiences during the COVID-19 pandemic, including a wide range of ongoing challenges related to resources, infrastructure, and available staff. Because unjust social determinants already affect urban Native populations disproportionately, special attention must be given to enhancing the fragile systems of services for these at-risk populations, and resources must be provided to address the most significant of these issues. Already under-resourced and over-burdened, UIO staff have adapted virtually every facet of their work at an intense and unrelenting pace, showing a degree of resilience reflective of the Native populations they serve. UIO staff in all roles are experiencing fatigue from the increased difficulty in conducting their jobs with increased responsibility, increased risk, and fewer resources. Mental health issues among staff are emerging and, in some cases, worsening and very few UIOs have the capacity to address these needs on top of other problems. Although UIO staff are attempting to adapt to a wide range of changes, their burden may be reduced by creating more efficient and streamlined communication channels and information dissemination internally and externally. Utilizing UIO staff as trusted community partners for outreach and community education could ensure receptivity and help eliminate doubts stemming from having received conflicting information from social media and other sources of misinformation.

## Data Availability Statement

The raw data supporting the conclusions of this article are not publicly available to protect participant confidentiality.

## Ethics Statement

Ethical review and approval was not required for the study on human participants in accordance with the local legislation and institutional requirements. Written informed consent for participation was not required for this study in accordance with the national legislation and the institutional requirements.

## Disclosure

This project was supported by the Centers for Disease Control and Prevention of the U.S. Department of Health and Human Services (HHS) as part of a financial assistance award totaling $1,500,000 with 100 percent funded by CDC/HHS. The contents are those of the author(s) and do not necessarily represent the official views of, nor an endorsement, by CDC/HHS, or the U.S. Government.

## Author Contributions

NC, JC, JI-A, and DA were all present for the listening sessions. NC served as primary facilitator throughout all listening sessions. JC served as a secondary facilitator. DA served as a notetaker. JI-A managed the chat and video functions while ongoing. NC and JC performed initial coding, data analysis and drafted the manuscript and report. JI-A contributed as an editor toward the finalization of the manuscript. All authors contributed to the article and approved the original submitted version.

## Conflict of Interest

The authors declare that the research was conducted in the absence of any commercial or financial relationships that could be construed as a potential conflict of interest.
